# Zika virus vertical transmission in children with confirmed antenatal exposure

**DOI:** 10.1038/s41467-020-17331-0

**Published:** 2020-07-14

**Authors:** Patrícia Brasil, Zilton Vasconcelos, Tara Kerin, Claudia Raja Gabaglia, Ieda P. Ribeiro, Myrna C. Bonaldo, Luana Damasceno, Marcos V. Pone, Sheila Pone, Andrea Zin, Irena Tsui, Kristina Adachi, Jose Paulo Pereira, Stephanie L. Gaw, Liege Carvalho, Denise C. Cunha, Leticia Guida, Mirza Rocha, James D. Cherry, Lulan Wang, Saba Aliyari, Genhong Cheng, Suan-Sin Foo, Weiqiang Chen, Jae Jung, Elizabeth Brickley, Maria Elisabeth L. Moreira, Karin Nielsen-Saines

**Affiliations:** 1 Fundação Oswaldo Cruz, Rio de Janeiro, Brazil; 2 David Geffen UCLA School of Medicine, Los Angeles, CA 90095 USA; 3 Biomedical Research Institute of Southern California, Oceanside, CA 92056 USA; 4 University of California, San Francisco School of Medicine, San Francisco, CA 94143 USA; 5 University of Southern California, Los Angeles, CA 90007 USA; 6 Department of Infectious Disease Epidemiology, London School of Hygiene and Tropical Medicine, London, WC1E 7HT UK

**Keywords:** Diseases, Infectious diseases, Viral infection

## Abstract

We report Zika virus (ZIKV) vertical transmission in 130 infants born to PCR+ mothers at the time of the Rio de Janeiro epidemic of 2015–2016. Serum and urine collected from birth through the first year of life were tested by quantitative reverse transcriptase polymerase chain reaction (PCR) and/or IgM Zika MAC-ELISA. Four hundred and seven specimens are evaluated; 161 sera tested by PCR and IgM assays, 85 urines by PCR. Sixty-five percent of children (*N* = 84) are positive in at least one assay. Of 94 children tested within 3 months of age, 70% are positive. Positivity declines to 33% after 3 months. Five children are PCR+ beyond 200 days of life. Concordance between IgM and PCR results is 52%, sensitivity 65%, specificity 40% (positive PCR results as gold standard). IgM and serum PCR are 61% concordant; serum and urine PCR 55%. Most children (65%) are clinically normal. Equal numbers of children with abnormal findings (29 of 45, 64%) and normal findings (55 of 85, 65%) have positive results, *p* = 0.98. Earlier maternal trimester of infection is associated with positive results (*p* = 0.04) but not clinical disease (*p* = 0.98). ZIKV vertical transmission is frequent but laboratory confirmed infection is not necessarily associated with infant abnormalities.

## Abstract (web summary)

Here, Brasil et al. investigate mother to child Zika virus (ZIKV) transmission rates in a large longitudinal cohort of pregnant ZIKV-positive women with their infants followed from the time of maternal infection through birth and onwards, finding high in utero transmission rates that do not predict clinical outcomes, suggesting follow-up of children with antenatal ZIKV exposure is necessary.

## Introduction

In 2015, Zika virus (ZIKV) reached Rio de Janeiro, Brazil, and local transmission of the virus was quickly confirmed^1^. At the time, our group had an ongoing study evaluating outcomes in pregnant women who developed a rash due to arboviral infections. With the onset of the ZIKV epidemic, we established a longitudinal cohort of pregnant women who presented with a rash within the prior 5 days and were found to be ZIKV positive in blood or urine by quantitative reverse transcriptase polymerase chain reaction (QRT-PCR) at the time of presentation^2^. We followed these women throughout pregnancy to delivery and reported on pregnancy outcomes^2^. Subsequently we reported outcomes of their children in the first months and years of life, including physical findings, neurologic examinations, neuroimaging results, complete eye exams, hearing assessments, and neurodevelopmental outcomes^2–7^.

Although all infants in this prospective Zika cohort were exposed in utero to maternal ZIKV infection, it is difficult to ascertain the true rate of ZIKV vertical transmission without infant laboratory results, as the majority of ZIKV-exposed children are not born with severe clinical features of congenital Zika syndrome^8^. Amniocentesis to detect ZIKV by RT-PCR in the amniotic fluid can confirm vertical transmission prenatally although this procedure is uncommonly performed in Brazil^7^. There is limited data on the sensitivity and specificity of testing after birth to confirm vertical transmission. In adults, a confirmed laboratory diagnosis of ZIKV is challenging, as there is a brief window period where virus detection in plasma or urine occurs^9^. Zika serologic testing has been further complicated in endemic areas by the cross-reactivity between ZIKV IgG antibodies and antibodies against dengue virus serotypes; in that scenario most surveillance systems rely on clinical criteria to identify cases^10,11^.

We sought to determine the utility of molecular and serologic testing in the diagnosis of mother-to-child-transmission of ZIKV. We report the frequency of ZIKV PCR and ZIKV-specific IgM detection in the serum and urine of children with confirmed prenatal ZIKV exposure. We also analyzed whether positive infant results for ZIKV were associated with abnormal pediatric outcomes.

## Results

### Study population

Our longitudinal cohort was comprised of 244 pregnant women with confirmed ZIKV infection during pregnancy, of whom 223 (91.4%) had live births. Of these, 216 infants had clinical follow-up beyond birth. In the early stages of the ZIKV epidemic in Rio de Janeiro between September 2015 to February 2016, ZIKV PCR and IgM detection assays were considered investigational and not diagnostic. For this reason, there was a delay in the collection of infant specimens while IRB approval for infant phlebotomy and urine collection was pending. As a result, from the original cohort of 216 infants, 130 children (60%) had blood and or urine specimens obtained for ZIKV detection. The present report focuses on these 130 infants with clinical follow-up with laboratory diagnostic evaluations. Table 1 reports clinical characteristics and timing of maternal infection for all 216 children in the cohort, with results stratified by those who received diagnostic testing and those who did not. As seen in the table, both groups were comparable, with the exception that untested children tended to have lower neurodevelopmental scores in the second to third years of life and all children with microcephaly were in the tested group.

**Table 1:** Frequency of abnormal findings among in utero ZIKV-exposed infants according to ZIKV postnatal testing status.

	All		Tested infants		Untested infants		*p*-Value*
	*N* = 216	%	*N* = 130	%	*N* = 86	%	
Infants with any abnormal findings	78/216	36.1	45/130	34.6	33/86	38.4	*p* = 0.70
Structural brain abnormalities	14/140	10.0	14/113	12.4	0/27	0	*p* = 0.13
Complete eye exam (abnormal)	9/137	6.6	6/109	5.5	3/28	10.7	*p* = 0.36
Hearing assessment (abnormal)	13/114	11.4	11/91	12.1	2/23	8.7	*p* = 0.68
Small for gestational age	10/216	4.6	10/130	7.7	0/86	0	*p* = 0.01
Below average neurodevelopment^a^	62/216	28.7	17/129	13.2	45/87	51.7	*p* < 0.001
1st Trimester maternal infection	54/216	25.0	40/130	30.7	14/86	16.3	*p* = 0.06
2nd Trimester maternal infection	109/21	50.5	61/130	46.9	48/86	55.8	*p* = 0.47
3rd Trimester maternal infection	53/216	24.5	29/130	22.3	24/86	27.9	*p* = 0.47
Microcephaly	8/216	3.7	8/130	6.1	0/86	0	*p* = 0.02

^a^Assessed through Bayley-III or HINES.

*Chi-square (Fisher’s exact *t*-test used when cell values equal zero).

### Diagnostic testing

**Fig. 1:**
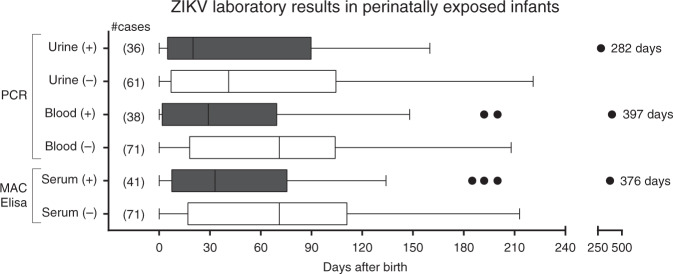
Timing of positive and negative ZIKV laboratory results over time by age. The figure reflects the number of assays performed in each assay category in specimens collected from 130 children. There were 161 serum specimens run for ZIKV PCR and ZIKV IgM and 85 urine specimens run for ZIKV PCR. The vertical line in the box represents the median, the box the interquartile range (10–90% of the data), and the whiskers the 95% confidence interval. The figure represents all the assays performed, not the number of children. All IgM assays were run once and in duplicate and all PCR assays were run once in triplicate. Children can be represented more than once. Error bars represent the standard deviation.

In total, 407 ZIKV diagnostic assays were run in specimens obtained from 130 exposed children. These included PCR assays in 161 serum and 85 urine samples, and 161 serum ZIKV IgM assays, as shown in Fig. 1. In total, 84 of 130 children (65%) had positive laboratory test results for ZIKV as seen in Table 2. The age at the time of performance of the first Zika diagnostic laboratory test ranged from birth to 148 days. Within the group of children tested in the first 90 days of life (*n* = 94), 70% (*N* = 66) tested positive by at least one detection method (39% positive by blood PCR, 48% by urine PCR, 39% by IgM). For 78 children who were tested beyond 90 days of age, including repeat testing, 33% were positive by any laboratory detection method, demonstrating that sensitivity of diagnostic testing for ZIKV dropped considerably beyond 3 months of age. This observation was mainly due to the fact that the sensitivity of serum detection assays (IgM and PCR) in identifying Zika infection declined after 3 months of age (from 39 to 15% for serum PCR and from 39 to 20% for IgM). Although fewer children were tested by urine PCR (73 versus 109 for serum PCR), urine was the most frequently positive detection method within any age group (49%), followed by Zika IgM (positivity rate of 37%) and Zika PCR of serum (35%) (Table 2).

**Table 2:** ZIKV vertical transmission by age and assay type.

	No. of children	Positive	%
Tested within the first 3 months of age	94 (72%)	66	70%
PCR serum	76 (81%)	30	39%
IgM	75 (80%)	29	39%
PCR urine	54 (57%)	26	48%
First tested after 3 months of age^a^	36 (28%)	18	50%
PCR serum	33 (92%)	7	21%
IgM	36 (100%)	7	19%
PCR urine	19 (53%)	8	42%
Tested after 3 months of age	78 (60%)	26	33%
PCR serum	62 (79%)	9	15%
IgM	65 (83%)	13	20%
PCR urine	23 (29%)	10	43%
All time points	130 (100%)	84	65%
PCR serum	109 (84%)	38	35%
IgM	112 (86%)	41	37%
PCR urine	73 (56%)	36	49%

^a^Ninety-four children had their first laboratory test for ZIKV infection within 90 days of age; 36 children had their first assay performed after 90 days of age. Some children had one laboratory assay performed prior to 90 days of age and a subsequent laboratory assay performed post 90 days of age. Any children in this situation would be counted in the table as tested prior to 90 days of age. If there was repeat testing after 90 days of age children are also counted in that category for that specific assay, i.e., the number of assays does not reflect the number of children, as some children had more than one assay.

A positive ZIKV laboratory result in antenatally exposed children was most frequently seen in the first 3 months of life (Table 2 and Fig. 1). Nevertheless, there were rare outliers within each assay category who tested positive beyond 200 days of age. Three children excreted the virus in urine at ages of 6.7, 8, and 9.4 months of age; none have developed abnormal clinical findings as of the time of this publication (3 years of age). However, two children with positive serum ZIKV PCR at ages 6 and 13 months were found to have abnormal funduscopic and hearing exams, respectively.

### Frequency of testing

The frequency of specimen collection is depicted in Table 3. The majority of children were tested only once in each assay category, although blood specimens were obtained more than once in over 1/3 of children. Only one patient tested positive twice on a later time point by the same diagnostic assay (Zika IgM). Among children who had repeated testing at later time points in the first year of life or beyond, the overwhelming trend was for patients to remain negative or switch from positive to negative results (Table 3). Nevertheless, two children went from negative to positive results by serum assays and three children had positive results in urine by PCR after two previous negative assays.

**Table 3:** Frequency of Zika testing (*n* = 407 in 130 children).

	No. of children	%	No. NEG every time	No. POS every time	No. changing from POS to NEG	No. changing from NEG to POS
Serum Zika RT-PCR^a^	109					
Once	67	61.5%	47	20		
Two time points	34	31.2%	21	0	11	2
Three time points	6	5.5%	3	0	3	0
Four time points	2	1.8%	0	0	1	1
Zika IgM Mac Elisa^b^	112					
Once	72	64.3%	52	20		
Two time points	32	28.6%	17	1	12	2
Three time points	7	6.3%	2	0	2	3
Four time points	1	0.9%	0	0	0	1
Urine Zika RT-PCR^c^	73					
Once	64	87.7%	34	30		
Two time points	6	8.2%	3	0	3	0
Three time points	3	4.1%	0	0	0	3

^a^Number of serum PCR tests (*n* = 161).

^b^Number of Zika IgM Mac Elisa (*n* = 161).

^c^Number of urine Zika RT-PCR (*n* = 85).

### Concordance of laboratory results

Supplementary Table 1 depicts the different diagnostic assay combinations and concordance between different assays before or after 90 days of life. The majority of children (82.3%) had two or three concurrent diagnostic assays at their first visit. Over all time points, IgM results were concordant with serum and/or urine PCR results 52.3% of the time, with a sensitivity of 65.5% and a specificity of 39.6% (Supplementary Table 1). Concordance up to 90 days of age was 52% with a sensitivity of 61.5% and a specificity of 37.1%. Beyond age 3 months concordance for IgM and PCR assays was 39%, sensitivity 31.5% and specificity 35.7%. Concordance between IgM and serum PCR results was higher, 61% for all assays performed, while concordance between serum and urine PCR results was 55% at all time points.

### Clinical assessments

Among the 130 children who were tested for ZIKV (Table 4), 113 (86%) had brain imaging, with 13% (*n* = 14) demonstrating structural brain abnormalities (8 cases of microcephaly and 6 others with severe brain abnormalities). Complete eye exams were performed in 109 children in this cohort (83.8%); 6 children (5.5%) had abnormal eye findings. Ninety-one children (70%) had hearing assessments with 11 abnormal results (12.1%). Twenty-five children were noted to have grossly abnormal neurologic exams in the first six months of life (19%). Ten children (7.7 %) were born small for gestational age reflecting in utero growth restriction. Neurodevelopmental evaluations performed after 1 year of age demonstrated that 17 of 129 children (13.2%) had severe developmental delay, 14 scored below 2 SD in one or more Bayley-III domains and 3 were abnormal by the HINES assessment. When all parameters were combined, 45 of 130 children (35%) had an abnormal finding in one or more of these clinical or neurodevelopmental assessments. Supplementary Table 2 provides detailed clinical data on children undergoing Bayley-III evaluations.

**Table 4:** Association between ZIKV laboratory results, clinical findings, and gestational age at infection.

		Any positive ZIKV-Lab result (*N* = 84)		Any negative ZIKV-Lab result (*N* = 46)		
	All infants	*n*	%	*n*	%	*p*-Value
All infants	130	84	65%	46	35%	
Infants with abnormal findings^a^	45	29	64%	16	36%	0.98
Infants with no abnormal findings^a^	85	55	65%	30	35%	
Brain Imaging	113	74	65%	39	35%	0.37
Structural abnormalities	14	11	79%	3	21%	
Fundoscopy	109	68	62%	41	38%	0.41
Abnormal	6	5	83%	1	17%	
Hearing Assessment	91	60	66%	31	34%	0.09
Abnormal	11	10	91%	1	9%	
Neurologic exam	130	84	65%	46	35%	0.94
Abnormal	25	16	64%	9	36%	
Gestational age assessment	130	84	65%	46	35%	0.74
Small for gestational age	10	6	60%	4	4%	
Neurodevelopment	129	83	64%	46	36%	0.76
Abnormal Bayley-III or HINES	17	12	71%	5	29%	
Gestational Trimester of Infection	130	84	65%	46	35%	
First	40	31	78%	9	23%	0.04
Second	61	39	64%	22	36%	
Third	29	14	48%	15	52%	

Pearson’s chi-square with Yates’ continuity correction was used unless expected cell count was <5, then Fisher’s exact test was used.

^a^Includes abnormal hearing, eye, gestational age size assessment, neurodevelopment, neurological exam, or structural brain abnormality.

### Laboratory confirmed ZIKV infection and clinical outcomes

**Fig. 2:**
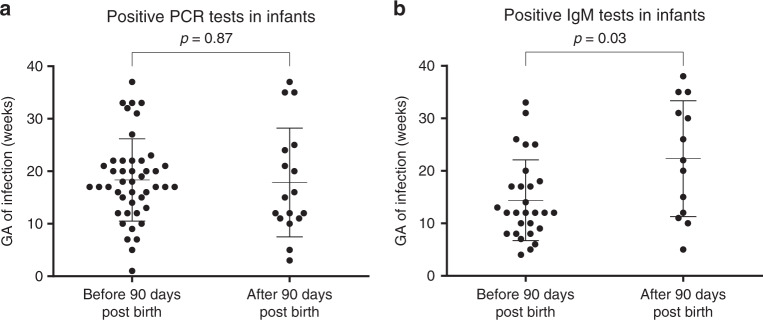
Infant positive PCR and IgM results by infant age and maternal gestational age of infection. Panel (**a**) depicts positive ZIKV PCR results in serum or urine for unique children. Before 90 days of age, 45 children had positive PCR results. After 90 days of age, 17 children had positive PCR results. The difference in distribution of positive PCR results between both age groups was not statistically significant, *p* = 0.87. Panel (**b**) depicts positive ZIKV IgM results. Before 90 days of infant age, 29 infants had positive results. After 90 days of age, 13 children had positive IgM results. The difference in distribution of positive IgM results between both age groups was statistically significant, *p* = 0.03. The statistical test used for comparison was a two-sided hypothesis Student T-test at the alpha 0.05 level. There were no adjustments for multiple comparisons. Error bars represent the standard deviation.

No statistical associations were identified between abnormal infant findings and positive ZIKV assay results, except for trimester of maternal infection. Infants born to mothers who contracted infection in the first trimester of pregnancy were more likely to have positive ZIKV PCR or IgM results (78%) as compared with those infected in the second (64%) or third trimesters (48%), *p* = 0.04 (Table 4). In addition, infants who were diagnosed by IgM in the first 90 days of life tended to have mothers who were infected earlier in gestation (median 14 weeks) than those diagnosed by IgM after 90 days of life (median 22 weeks), *p* < 0.01, as seen in Fig. 2. The median week of infection during gestation did not differ between infants diagnosed by PCR in blood or urine before or after 90 days of age (median 18 weeks for both groups), *p* = 0.8470.

Although no statistical association was identified between clinical abnormalities and a positive ZIKV test result (potentially because of the relatively small numbers in each category), we saw a tendency for specific abnormalities to cluster among children with positive results. Five of six infants with abnormal eye exams (83%), 11 of 14 with structural brain abnormalities (92%), and 10 of 11 with hearing deficits (91%) had a positive diagnostic assay for ZIKV infection. However, this clustering was not as clearly noted in children with other clinical findings such as developmental delay (12 of 17, or 71%), abnormal neurologic exams (16 of 25, 64%), or who were small for gestational age (6 of 10, 60%). None of these findings, however, achieved statistical significance as noted in Table 3. Overall, 35% of the 130 children tested for ZIKV infection had at least one abnormal finding as seen in Table 3 which means the majority of the cohort (65%) had a normal outcome. Any positive laboratory test was found in 29 of 45 children (64%) with abnormal findings. Conversely, 55 of 85 children with no abnormalities (65%) also tested positive for ZIKV, demonstrating that a positive ZIKV test result was found in 2/3 of children with either normal or abnormal clinical findings.

## Discussion

Laboratory confirmation of ZIKV infection is challenging due to the short window of viremia and viruria enabling PCR detection, and also because of the serologic cross-reactivity between Zika and dengue viruses. In prenatally exposed children, a laboratory diagnosis of ZIKV infection is even more challenging. The duration of ZIKV viremia and viruria in congenitally infected children is unknown, and it is unclear if infants infected very early during intrauterine life have detectable virus at birth, as the duration of viral shedding from intrauterine infection has not been described. In addition, it is unclear if viral presence in blood and/or urine in congenitally infected children is constant or intermittent. It is unknown whether all children infected with ZIKV in utero form adequate antibody responses easily detectable by IgM assays. For example, experience from other congenital infections (such as rubella and CMV) tells us that IgM assays are suboptimal for diagnosis of viruses that affect T and B cell function. Nevertheless, IgM does not cross the placenta, so we therefore surmise that positive serologic results would reflect an infant’s prior exposure to the virus. In the present cohort, cross-reactivity with dengue virus would be unlikely, as at the time infant specimens were obtained in Rio de Janeiro (2105–2017) there was absent to minimal circulation of dengue viruses.

The prospective nature of the cohort, with infants followed from the time of maternal infection, through birth, and onward allows us a unique opportunity for evaluation of laboratory confirmed ZIKV congenital infection rates. Sixty-five percent of children in our cohort had laboratory evidence of ZIKV infection, including a large proportion of children who were infected in the first trimester of pregnancy (78%). Interestingly, having a positive ZIKV laboratory result did not necessarily correlate with infant outcomes; this could be due to limitations of the present sample size or a real phenomenon. What we can conclude from our study is that ZIKV testing of infants does not necessarily correlate with clinical findings, particularly in asymptomatic children. Anecdotally, in our cohort and that of other Zika cohorts in Brazil, there were symptomatic children tested in the first 48 h of life who did not have detectable virus in either blood or urine. Potentially these children were infected so early during pregnancy that viral infection is gone by the time of birth and only the sequelae of infection is present. This is noted in other congenital infections such as congenital varicella syndrome^12^. If infection is later in pregnancy, viral shedding may be more frequently seen, but the teratogenic sequelae will not be as obvious as the development of the neurologic system in the first 12 weeks of gestational age is past. So viral detection may not clearly correlate with the presence of clinical findings over time. This phenomenon is also seen in some children with congenital CMV^13^. Children who acquire rubella after 20 weeks of gestation also do not have findings of congenital rubella syndrome^14^. The high rate of positive laboratory results demonstrating infant ZIKV infection cannot be attributable to a high number of symptomatic children in the cohort. Eighty-five of 130 children in the study (65%) did not have any abnormal clinical manifestations at their last medical visit and did not have below average neurodevelopmental evaluations between ages 2 to 3 years. An equal proportion of children with both normal and abnormal findings tested positive (64% and 65%, respectively). We conclude that ZIKV has a very high in utero transmission rate but laboratory confirmed ZIKV infection in an infant did not equate to the presence of severe congenital abnormalities in our cohort.

The transmission rate reported in our study is considerably higher than the transmission rate of 26% reported by colleagues in the French Guiana Zika infant cohort^15^. In that study, ZIKV-exposed children were clinically evaluated and tested for ZIKV in the first 7 days of life. Because the degree of intermittent viral shedding in the blood and urine in ZIKV infected infants has not been well characterized, testing for ZIKV over a longer period of time during infancy likely enhances detection of positive results. One study of pregnant women returning from epidemic areas to New York evaluated infant diagnosis around the time of birth, however, the majority of maternal cases were suspected rather than PCR-confirmed infections, with a 7% vertical transmission rate at birth for ZIKV infection reported in that study^16^. Differences in study design (retrospective versus prospective, follow-up time, sampling, study population and definitions of infection, i.e., confirmed versus suspected) make it difficult to compare results across different studies.

Our specimens were collected and patients were recruited during the ZIKV epidemic, before there were any guidelines for ZIKV diagnostic evaluation of infants, including current CDC guidelines^17^. One could argue that the large number of positives could result from postnatal exposure to ZIKV. However, we should consider that all children in the present study had PCR-confirmed ZIKV in utero exposure, so they were all at high risk of contracting the virus. This differs significantly from scenarios in which the maternal diagnosis of Zika infection is unconfirmed and infant exposure status is unknown. Because ZIKV diagnostics in infants was considered investigational at the time, we did not collect specimens from infants born early on in the epidemic, which is when ZIKV was still circulating, which is a study limitation, as collection of early specimens in all children would have been ideal. In May 2016 we saw a dramatic decline in ZIKV circulation in Rio de Janeiro, which coincided with a Chikungunya outbreak^18,19^. For this reason, it is unlikely that the tested group of infants had a high chance of acquiring postnatal ZIKV infection, as by the time most of them were born, the virus was no longer circulating in Rio de Janeiro. We nevertheless did observe a much higher frequency of positive results in the first 3 months of life, reflecting a short window for detection of congenitally acquired ZIKV infection, including a short period of ZIKV IgM positivity, a finding that has been reported in adults following ZIKV infection^20^.

One of our study limitations is that we did not perform sequential testing of all infants at regular time points. We were able to perform PCR immediately following specimen collection for most infant serum and urine specimens which contributes to a greater sensitivity of the assay, as PCR identification of ZIKV tends to greatly diminish with freezing and thawing of specimens^21^. Nevertheless, because sequential, methodical testing at regular time points was not possible for many children, we cannot ascertain that the ones with negative results were true negatives. In addition, we did not have as many early postnatal specimens available for testing to assure ascertainment of infection in infants born early on in the epidemic (when IRB approval was pending) and this is a study limitation. We certainly could have missed the window of positivity for a number of children who tested PCR negative for ZIKV subsequently. Another study limitation is that we did not have a control group of ZIKV unexposed children of the same age and from the same location for comparison of clinical neurodevelopmental outcomes and serial laboratory testing. Because ZIKV infection can be asymptomatic during pregnancy, we were unable to recruit an equal number of children in parallel in whom we could effectively rule out past maternal ZIKV infection during gestation. In Rio de Janeiro during the epidemic, any pregnant woman could have silent ZIKV infection, while infants born without clinical features of congenital Zika would not be identified as having in utero exposure. Therefore, we could not rule out that control children were in truth controls. It is also unclear if all children exposed to ZIKV are capable of developing a robust IgM response. We know that in other congenital infections such as rubella and CMV, there can be a significant delay in the development of IgM antibodies^13,14^. In this sense, we believe the 65% ZIKV transmission rate is likely an underestimate.

We found a statistically significant association between earlier maternal infection in pregnancy and ZIKV infant infection, which demonstrates that transmission events are more likely when women are infected earlier in pregnancy. Nevertheless, close to 50% of women infected in the third trimester also had infants with positive results, which underscores that ZIKV is highly transmissible throughout the entire gestational period. We also observed that women infected earlier in pregnancy tended to have infants with positive IgM results earlier in life. Conversely, women infected later in pregnancy tended to have infants whose IgM results were positive beyond 90 days of life. One caveat is that all women in our cohort were symptomatic. In a prior analysis we did not find any associations between the magnitude of maternal symptoms and infant clinical findings^22^. In addition, congenital Zika syndrome has been described in children of asymptomatic women^8^. Potentially women with symptomatic ZIKV infection could have children with more clinical findings, however, this hypothesis has not yet been confirmed. Future research to determine factors related to transplacental transmission of the virus over gestation is needed.

As is the case with most congenitally acquired viral infections, such as CMV, rubella, or HIV, PCR of urine and serum (combined) was superior in diagnosing ZIKV infection as compared to serology alone. Urine PCR appeared to be most sensitive yielding the highest proportion of positive results^23^. As urine specimens were collected in 56% of the children in the cohort, higher rates of vertical transmission might have been observed if more urine specimens were obtained. IgM and serum PCR tended to provide similar rates of positivity. Nevertheless, because we did not have sequential, simultaneous testing of specimens at regular intervals by all assays, comparison between types of specimens and frequency of positive and negative results should be interpreted with caution.

Interestingly, a small number of children had positive PCR results after 200 days of life. One child developed positive IgM results during the same time period. Although we cannot rule out postnatal infection, because ZIKV stopped circulating in Rio de Janeiro by that time, postnatal infection seems unlikely. ZIKV could be shed intermittently in the blood or urine, similarly to congenital CMV or congenital rubella^13,14^. These children had negative test results before, which would suggest intermittent shedding. A delayed IgM response could be present in congenitally acquired ZIKV infection, explaining late positive IgM results. Intermittent and long-term shedding of ZIKV in the urine is very concerning for the presence of viral reservoirs with low-level replication that persist in some neonates.

A negative laboratory test result for ZIKV infection early in life will not rule out congenitally acquired infection because in many cases the narrow window period for testing may be missed. A positive result, however, particularly in our setting where women had PCR-confirmed infection is helpful in ascertaining infection versus exposure. Current CDC testing guidelines recommend testing of infants as early as possible, preferably within the first few days after birth^13^, although according to our results testing specimens within the first few weeks up to 3 months of age might still be useful. Distinguishing between congenital, perinatal, and postnatal infection is difficult in infants living in endemic areas who are not tested soon after birth. Nevertheless, one has to take into account the epidemiologic surveillance data to ascertain if circulation of the virus is ongoing when interpreting results. There are no studies to date, to our knowledge, reporting data on sequential infant testing following PCR-confirmed ZIKV antenatal exposure. Whether the pattern of viral shedding in antenatally infected infants is persistent or intermittent is unknown.

In summary, approximately 2/3 of children tested for ZIKV infection in our prospective cohort were positive, which means ZIKV in utero transmission is very frequent. Having a positive test result is not necessarily associated with a bad outcome, but there is an association with earlier maternal infection in pregnancy. Given the high transmission rate and the fact that a negative laboratory assay for ZIKV may not rule out infection, all infants with documented or potential antenatal ZIKV exposure should be followed long term. Intermittent viral shedding in the urine occurs in a small number of infants and suggests the presence of viral reservoirs. We have seen that a normal early infant assessment may not necessarily guarantee normal neurodevelopment or absence of sensory dysfunction^5^. As laboratory diagnostic testing does not seem to predict infant outcomes, close follow-up of all children with antenatal ZIKV exposure should be the norm.

## Methods

### Study population

The study population was comprised by infants born to women enrolled in a longitudinal cohort of confirmed ZIKV infection during pregnancy^2^, for whom postnatal samples of blood and/or urine were collected for ZIKV laboratory diagnosis. Gestational age of infection was estimated based on the day the pregnant woman first presented with the rash due to ZIKV infection confirmed by PCR in blood or urine. All children were followed at the Fundação Oswaldo Cruz (Fiocruz) in Rio de Janeiro and were enrolled from December 2015 to December 2016.

### ZIKV PCR detection

Infant serum and urine specimens were obtained following parental signed informed consent. Serum was collected by standard phlebotomy procedures and processed immediately for PCR while additional aliquots were stored at −80 °C for subsequent testing for Zika IgM. Urine specimens collected by bagged urine collection, spun in a refrigerated centrifuge for 10 min at 700 × *g*, and the supernatant was aliquoted and processed immediately for PCR. Both serum and urine specimens were tested by qRT-PCR amplification assay for ZIKV in triplicate using the TaqMan probe (Applied Biosystems) for detection and absolute quantification of ZIKV^2,11^. The following primers were used [Primer Genome Position Sequence (5′ → 3′)]:

ZIKV 1086 1086–1102 CCGCTGCCCAACACAAG.

ZIKV 1162c 1162–1139 CCACTAACGTTCTTTTGCAGACAT.

ZIKV 1107-FAM 1107–1137 AGCCTACCTTGACAAGCAGTCAGACACTCAA.

Laboratory testing sites included the research laboratory of the Instituto Fernandes Figueira Hospital (IFF) and the Laboratory of Molecular Biology of Flaviviruses, Fiocruz.

### ZIKV IgM detection

ZIKV serologic testing of infants was performed in duplicate serum aliquots using IgM antibody capture Zika MAC-ELISA from the Centers for Disease Control and Prevention (CDC, Fort Collins, CO, EUA) according to manufacturer instructions^24^. All serum samples were diluted at 1:400 in wash buffer and conjugate antibodies were diluted at 1:2500 in blocking buffer.

### Pediatric outcomes

Pediatric outcomes (normal versus abnormal) were determined based on specific clinical findings.

#### Early infant findings

Were defined based on the presence of any of the following medical findings in the first 3 months of life: (1) Microcephaly (MC) defined as head circumference *Z*-score <−2 (moderate) and <−3 (severe)^25^; (2) small for gestational age (SGA) at birth based on sex-specific curves by Intergrowth-21^26^; (3) abnormal eye findings following a complete exam with funduscopic evaluation performed by pediatric ophthalmologists^27–30^; (4) abnormal hearing assessments evaluated through brainstem evoked response audiometry (BERA)^3,4^; (5) very abnormal neurological exam, with a constellation of findings on repeated physical examination including a combination of hyper- or hypotonia, clonus, contractures/arthrogryposis, seizures, continuous irritability (inconsolable crying)^2–6^; abnormal finding on neuroimaging through transfontanelle ultrasounds, and/or computerized tomography, and/or magnetic resonance imaging with identification of structural brain abnormalities (i.e., disorders altering normal brain morphology)^2,31^.

#### Later neurodevelopmental findings

Defined as abnormal based on the presence of any of the following beyond 6 months of life: (1) Severe developmental delay with a Bayley-III Score^32^ under −2SD (<70) in any functional domain (cognitive, language or motor)^3,4^; (2) abnormal time to achievement of developmental stages through the Hammersmith Infant Neurological Examination scheme (HINES), evaluating neurological exam, motor function and state of behavior^3,4^.

### Statistical analysis

Chi-square tests were used to examine differences in categorical covariates between groups. When cell counts were <5 infants for any group, Fisher-exact tests were performed. Positive and negative predictive values were calculated for below normal development based on clinical outcomes. Associations between gestational age at infection and clinical outcomes including microcephaly, structural image abnormalities, hearing or eye abnormalities, development, or ZIKV laboratory results were explored by Pearson’s chi-square. Analyses were conducted using the statistical package R (R version 3.0.1, The R Foundation for Statistical Computing, www.r-project.org).

### Study oversight

The study was approved by the institutional review boards of the Fundação Oswaldo Cruz (Fiocruz) and the University of California, Los Angeles. Parents or guardians provided written informed consent.

### Reporting summary

Further information on research design is available in the Nature Research Reporting Summary linked to this article.

## Supplementary information

Supplementary Information

Reporting Summary

## Data Availability

The authors declare that the data supporting the findings of this study are available within the article and its Supplementary Information files, or are available from the authors upon request. Source data are provided with this paper.

## Source data

Source Data
