# Supplementary material for: Zika virus vertical transmission in children with confirmed antenatal exposure
Source: Nat Commun. 2020 Jul 14;11:3510. doi: 10.1038/s41467-020-17331-0 (PMC7360785; doi:10.1038/s41467-020-17331-0)
Supplement: Supplementary file 1 — Supplementary Information [file 41467_2020_17331_MOESM1_ESM.pdf]

Supplementary Information

Zika Virus Vertical Transmission in Children with Confirmed Antenatal Exposure.

Brasil et al.

Supplementary Table 1: Distribution of diagnostic testing per individual children and concordance of test results

All Time points (Children)			Before 90 days		After 90 days	
	N=130	%	N=94	%	N=78	%
PCR serum	1	0·8%	1	1·1%	2	2·6%
IgM serum	5	3·8%	1	1·1%	4	5·1%
PCR urine	16	12·3%	17	18·1%	11	14·1%
PCR serum and IgM	52	40·0%	38	40·4%	49	62·8%
PCR serum + PCR urine	1	0·8%	1	1·1%	0	0·0%
PCR urine, IgM	0	0·0%	0	0·0%	1	1.3%
PCR serum, urine, IgM	55	42·3%	36	38·3%	11	14·1%

Concordance of diagnostic test results

All time points (Assays)			Assays performed before 90 days of life		Assays performed after 90 days of life	
Blood PCR result	IgM Result		IgM Result		IgM Result	
	NEG	POS	NEG	POS	NEG	POS
NEG	47	24	29	17	43	8
POS	21	15	17	11	5	4
Blood PCR result	PCR urine results		PCR urine results		PCR urine results	
	NEG	POS	NEG	POS	NEG	POS
NEG	21	16	16	7	4	4
POS	9	10	6	8	2	1
IgM result	PCR urine results		PCR urine results		PCR urine results	
	NEG	POS	NEG	POS	NEG	POS
NEG	13	17	8	10	4	5
POS	17	9	14	4	2	1
IgM result	Serum and urine PCR results		Serum and urine PCR results		Serum and Urine PCR results	
	NEG	POS	NEG	POS	NEG	POS
NEG	36	32	24	22	39	9
POS	19	21	15	13	8	5
Concordance between IgM and PCR assays	52·3%		51·5%		39·0%	
Concordance between IgM and serum PCR	61·3%		54·1%		78·3%	
Concordance between Serum and urine PCR	55·4%		64·9%		45·5%	
Sensitivity	65·5%		61·5%		83·0%	
Specificity	39·6%		37·1%		35·7%	
Positive predictive value (PPV)	52·9%		52·2%		81·3%	
Negative predictive value (NPV)	52·5%		46·4%		38·5%	

* Sensitivity, specificity, PPV and NPV are based on any positive PCR as the gold standard compared to IgM

Supplementary Table 2

Clinical Characteristics of Children who underwent ZIKV Laboratory Testing and Bayley-3 Evaluations (N=115)

Gestational Age Infection	Gestational Age Birth	Microcephaly	SGA	Premature	Eye Exam	Hearing Exam	CNS Imaging	Bayley-3 Age	Cognitive Score	Language Score	Motor Score
20	37	NO	NO	NO	-	-	-	12	100	77	118
12	34	NO	NO	YES	NORMAL	NORMAL	ABNORMAL	14	95	71	97
15	35	NO	NO	YES	NORMAL	ABNORMAL	ABNORMAL	25	80	86	88
37	37	NO	NO	NO	NORMAL	NORMAL	NORMAL	25	90	89	91
20	37	NO	NO	NO	NORMAL	NORMAL	NORMAL	25	90	91	91
22	38	NO	NO	NO	NORMAL	NORMAL	NORMAL	12	100	89	97
22	38	NO	NO	NO	NORMAL	NORMAL	ABNORMAL	18	105	94	103
17	39	NO	NO	NO	NORMAL	NORMAL	NORMAL	25	110	94	97
17	38	NO	NO	NO	-	-	NORMAL	13	125	118	100
32	39	NO	NO	NO	NORMAL	NORMAL	NORMAL	19	100	109	97
26	38	NO	NO	NO	NORMAL	NORMAL	ABNORMAL	19	85	91	79
35	41	NO	NO	NO	NORMAL	NORMAL	NORMAL	24	100	94	107
12	39	NO	NO	NO	NORMAL	NORMAL	NORMAL	25	95	91	97
25	37	NO	YES	NO	NORMAL	NORMAL	ABNORMAL	28	95	86	94
6	39	NO	NO	NO	NORMAL	-	NORMAL	18	70	62	94
12	39	NO	NO	NO	NORMAL	-	NORMAL	19	105	91	110
12	38	NO	NO	NO	NORMAL	NORMAL	ABNORMAL	13	95	91	103
17	35	NO	NO	YES	NORMAL	NORMAL	NORMAL	19	85	89	79
10	40	NO	NO	NO	NORMAL	NORMAL	NORMAL	25	100	77	100
17	34	NO	NO	YES	NORMAL	ABNORMAL	ABNORMAL	14	105	83	88
17	34	NO	NO	YES	NORMAL	-	NORMAL	14	100	91	97
16	37	NO	NO	NO	NORMAL	-	ABNORMAL	22	95	112	107
17	38	NO	NO	NO	NORMAL	NORMAL	NORMAL	29	90	89	88
12	38	NO	NO	NO	NORMAL	NORMAL	NORMAL	18	105	106	103
8	38	NO	NO	NO	NORMAL	NORMAL	NORMAL	25	85	86	91
11	38	NO	NO	NO	NORMAL	NORMAL	ABNORMAL	12	95	89	79

14	39	NO	NO	NO	NORMAL	NORMAL	ABNORMAL	18	90	62	94
37	39	NO	NO	NO	NORMAL	NORMAL	NORMAL	18	115	86	107
10	38	YES	NO	NO	NORMAL	NORMAL	ABNORMAL	24	55	56	46
12	37	NO	NO	NO	-	-	NORMAL	9	105	112	118
35	40	NO	NO	NO	NORMAL	NORMAL	NORMAL	24	90	79	91
11	38	NO	NO	NO	NORMAL	NORMAL	NORMAL	26	115	115	110
20	34	NO	YES	YES	NORMAL	NORMAL	NORMAL	32	110	91	88
19	38	NO	NO	NO	NORMAL	NORMAL	ABNORMAL	18	90	86	82
22	39	NO	NO	NO	NORMAL	NORMAL	ABNORMAL	12	95	94	103
16	38	NO	NO	NO	NORMAL	-	NORMAL	20	90	83	79
30	39	NO	NO	NO	-	NORMAL	NORMAL	20	130	103	112
24	37	NO	NO	NO	NORMAL	NORMAL	ABNORMAL	19	100	79	107
27	35	NO	NO	YES	NORMAL	NORMAL	ABNORMAL	26	85	65	76
16	37	NO	NO	NO	NORMAL	-	-	12	95	100	110
5	40	NO	YES	NO	-	ABNORMAL	ABNORMAL	12	120	91	103
11	38	NO	NO	NO	NORMAL	-	ABNORMAL	12	105	94	103
12	36	NO	NO	YES	-	-	NORMAL	18	75	74	88
6	39	NO	NO	NO	NORMAL	NORMAL	NORMAL	25	90	71	100
39	38	NO	NO	NO	NORMAL	NORMAL	NORMAL	20	100	91	94
34	38	NO	NO	NO	NORMAL	NORMAL	NORMAL	18	95	97	110
16	39	NO	YES	NO	NORMAL	NORMAL	ABNORMAL	20	85	94	94
4	34	NO	NO	YES	NORMAL	NORMAL	NORMAL	24	65	47	76
22	41	NO	NO	NO	NORMAL	NORMAL	NORMAL	12	120	97	110
12	38	NO	NO	NO	-	-	NORMAL	18	105	100	103
16	38	NO	NO	NO	NORMAL	-	-	19	100	97	97
10	39	YES	NO	NO	NORMAL	-	ABNORMAL	24	55	55	55
17	41	NO	NO	NO	NORMAL	NORMAL	ABNORMAL	12	115	86	94
14	39	NO	NO	NO	-	NORMAL	NORMAL	25	90	65	107
11	36	NO	NO	YES	NORMAL	NORMAL	NORMAL	13	95	77	97
29	38	NO	NO	NO	-	-	NORMAL	18	100	91	97
33	34	NO	NO	YES	NORMAL	ABNORMAL	NORMAL	18	100	71	107
33	34	NO	NO	YES	NORMAL	ABNORMAL	NORMAL	18	95	65	103
21	39	NO	NO	NO	NORMAL	NORMAL	ABNORMAL	25	95	89	97

19	36	NO	NO	YES	NORMAL	NORMAL	NORMAL	18	100	103	115
38	39	NO	NO	NO	NORMAL	-	ABNORMAL	19	115	74	107
23	40	NO	YES	NO	NORMAL	NORMAL	NORMAL	19	90	91	76
18	38	NO	NO	NO	NORMAL	-	ABNORMAL	12	115	100	94
7	38	NO	NO	NO	NORMAL	NORMAL	NORMAL	14	120	91	110
20	38	NO	NO	NO	NORMAL	NORMAL	ABNORMAL	13	105	89	85
35	40	NO	NO	NO	NORMAL	NORMAL	NORMAL	18	85	83	82
26	35	NO	NO	YES	-	ABNORMAL	-	28	100	91	94
32	41	NO	NO	NO	NORMAL	NORMAL	NORMAL	11	105	100	110
18	37	NO	NO	NO	NORMAL	ABNORMAL	ABNORMAL	28	95	77	88
12	38	YES	NO	NO	NORMAL	NORMAL	ABNORMAL	13	105	103	85
21	40	NO	NO	NO	NORMAL	NORMAL	NORMAL	25	85	65	88
8	36	NO	NO	YES	NORMAL	-	NORMAL	17	110	94	107
27	39	NO	NO	NO	NORMAL	NORMAL	NORMAL	13	120	97	103
23	40	NO	NO	NO	NORMAL	NORMAL	NORMAL	18	85	91	85
18	37	NO	NO	NO	NORMAL	NORMAL	NORMAL	18	85	74	79
8	39	YES	NO	NO	ABNORMAL	-	ABNORMAL	24	55	55	55
27	41	NO	NO	NO	NORMAL	NORMAL	NORMAL	18	105	100	107
32	40	NO	NO	NO	NORMAL	NORMAL	NORMAL	13	110	100	97
30	40	NO	NO	NO	NORMAL	ABNORMAL	-	18	105	103	112
25	40	NO	NO	NO	NORMAL	-	NORMAL	18	95	106	110
26	40	NO	NO	NO	NORMAL	NORMAL	NORMAL	27	105	97	112
10	38	NO	NO	NO	NORMAL	NORMAL	NORMAL	20	105	100	110
5	38	NO	NO	NO	NORMAL	NORMAL	NORMAL	17	100	83	103
20	38	NO	NO	NO	NORMAL	NORMAL	ABNORMAL	13	105	91	88
12	35	NO	YES	YES	NORMAL	NORMAL	NORMAL	14	80	71	70
13	40	YES	YES	NO	ABNORMAL	ABNORMAL	ABNORMAL	24	55	55	55
31	37	NO	NO	NO	NORMAL	-	NORMAL	21	90	91	97
3	39	NO	NO	NO	-	-	-	22	90	89	97
7	38	NO	NO	NO	NORMAL	NORMAL	ABNORMAL	13	105	79	97
22	41	NO	NO	NO	NORMAL	NORMAL	ABNORMAL	25	90	79	88
22	39	NO	NO	NO	NORMAL		NORMAL	14	110	83	100
10	40	NO	NO	NO	NORMAL	NORMAL	NORMAL	18	90	97	82

8	39	NO	NO	NO	-	NORMAL	-	15	95	115	107
21	38	NO	NO	NO	ABNORMAL	NORMAL	ABNORMAL	26	100	91	97
23	39	NO	NO	NO	-	-	-	7	110	106	97
20	40	NO	NO	NO	NORMAL	NORMAL	NORMAL	12	120	77	112
33	39	NO	NO	NO	NORMAL	NORMAL	NORMAL	23	85	94	97
9	40	YES	NO	NO	ABNORMAL	ABNORMAL	ABNORMAL	24	55	55	55
27	40	NO	NO	NO	NORMAL	NORMAL	NORMAL	18	90	91	85
25	38	NO	NO	NO	NORMAL	NORMAL	NORMAL	28	120	91	91
30	31	NO	NO	YES	NORMAL	-	NORMAL	25	85	76	77
35	38	NO	NO	NO	-	-	-	12	100	77	91
22	34	YES	YES	YES	ABNORMAL	-	ABNORMAL	24	55	55	55
8	38	NO	NO	NO	NORMAL	-	NORMAL	27	85	80	88
15	38	NO	NO	NO	NORMAL	NORMAL	NORMAL	25	85	91	100
15	40	YES	YES	NO	NORMAL	-	NORMAL	19	100	89	103
15	41	NO	NO	NO	NORMAL	ABNORMAL	NORMAL	22	95	97	94
7	39	NO	NO	NO	ABNORMAL	NORMAL	NORMAL	12	115	83	103
29	40	NO	NO	NO	NORMAL	NORMAL	NORMAL	24	90	91	88
20	37	NO	NO	NO	NORMAL	NORMAL	NORMAL	16	105	89	103
4	40	NO	NO	NO	NORMAL	NORMAL	NORMAL	18	105	62	103
1	38	NO	NO	NO	NORMAL	NORMAL	NORMAL	24	95	86	97
28	39	NO	YES	NO	NORMAL	NORMAL	NORMAL	13	115	106	100
12	39	NO	NO	NO	-	NORMAL	-	18	105	79	103
24	40	NO	NO	NO	NORMAL	-	-	20	85	103	91

SGA: Small for gestational age; CNS: Central nervous system

OBS: Children who only underwent Hammersmith Infant Neurologic Examination (HINES) are not included (N=14)
