# Supplementary material for: Zika virus vertical transmission in children with confirmed antenatal exposure
Source: Nat Commun. 2020 Jul 14;11:3510. doi: 10.1038/s41467-020-17331-0 (PMC7360785; doi:10.1038/s41467-020-17331-0)
Supplement: Supplementary file 2 — Reporting Summary [file 41467_2020_17331_MOESM2_ESM.pdf]

nature research

Corresponding author(s): Karin Nielsen-Saines, MD, MPH

Last updated by author(s): 6/15/2020

Reporting Summary

Nature Research wishes to improve the reproducibility of the work that we publish. This form provides structure for consistency and transparency in reporting. For further information on Nature Research policies, see Authors & Referees and the Editorial Policy Checklist.

Statistics

For all statistical analyses, confirm that the following items are present in the figure legend, table legend, main text, or Methods section.

n/a	Confirmed	
☐	☒	The exact sample size (*n*) for each experimental group/condition, given as a discrete number and unit of measurement
☐	☒	A statement on whether measurements were taken from distinct samples or whether the same sample was measured repeatedly
☐	☒	The statistical test(s) used AND whether they are one- or two-sided<br>*Only common tests should be described solely by name; describe more complex techniques in the Methods section.*
☐	☒	A description of all covariates tested
☐	☒	A description of any assumptions or corrections, such as tests of normality and adjustment for multiple comparisons
☐	☒	A full description of the statistical parameters including central tendency (e.g. means) or other basic estimates (e.g. regression coefficient) AND variation (e.g. standard deviation) or associated estimates of uncertainty (e.g. confidence intervals)
☐	☒	For null hypothesis testing, the test statistic (e.g. *F*, *t*, *r*) with confidence intervals, effect sizes, degrees of freedom and *P* value noted<br>*Give P values as exact values whenever suitable.*
☒	☐	For Bayesian analysis, information on the choice of priors and Markov chain Monte Carlo settings
☒	☐	For hierarchical and complex designs, identification of the appropriate level for tests and full reporting of outcomes
☐	☒	Estimates of effect sizes (e.g. Cohen's *d*, Pearson's *r*), indicating how they were calculated

Our web collection on statistics for biologists contains articles on many of the points above.

Software and code

Policy information about availability of computer code

Data collection	Data was collected from chart abstraction of patient medical records and recorded into Microsoft Access. Data was coded using R, version 3.0.1 from the R Foundation for Statistical Computing.
Data analysis	R, version 3.0.1 from the R Foundation for Statistical Computing was used for data analysis. Code was dervived from the following R libraries: Base, epibasix, car, xlsx, pastecs, psych, ggplot2, and lme4.

For manuscripts utilizing custom algorithms or software that are central to the research but not yet described in published literature, software must be made available to editors/reviewers. We strongly encourage code deposition in a community repository (e.g. GitHub). See the Nature Research guidelines for submitting code & software for further information.

Data

Policy information about availability of data

All manuscripts must include a data availability statement. This statement should provide the following information, where applicable:

- Accession codes, unique identifiers, or web links for publicly available datasets
- A list of figures that have associated raw data
- A description of any restrictions on data availability

TThe data supporting the findings of this study are available within the paper and in the supplementary information files (Supplementary Tables 1 and 2).

Field-specific reporting

Please select the one below that is the best fit for your research. If you are not sure, read the appropriate sections before making your selection.

☐ Life sciences ☒ Behavioural & social sciences ☐ Ecological, evolutionary & environmental sciences

Behavioural & social sciences study design

All studies must disclose on these points even when the disclosure is negative.

Study description	This was a prospective study which followed infants born to women enrolled in a longitudinal cohort of confirmed ZIKV infection during pregnancy for whom postnatal samples of blood and/or urine were collected for Zika virus (ZIKV) laboratory diagnosis. All children were followed at the Fundação Oswaldo Cruz (Fiocruz) in Rio de Janeiro and were enrolled between December 2015 to December 2016. Infant serum and urine specimens were obtained from birth throughout the first year of life and tested by quantitative reverse transcriptase polymerase chain reaction (PCR) and/or the presence of IgM (Zika MAC-ELISA, CDC). The aim of the study was to report ZIKV vertical transmission rates in children with antenatal ZIKV exposure. A total of 130 infants were enrolled. Four hundred and seven specimens were evaluated; 161 sera were tested by PCR and IgM assays, 85 urines by PCR. Sixty-five percent of children (N=84) were positive in at least one assay. Of 94 children tested within 3 months of age, 70% were positive. Positivity declined to 33% after 3 months. Five children were PCR+ beyond 200 days of life. Concordance between IgM and PCR results was 52%, sensitivity 65%, specificity 40% (positive PCR results as gold standard). IgM and serum PCR were 61% concordant; serum and urine PCR 55%. Most children (65%) were clinically normal. Equal numbers of children with abnormal findings (29 of 45, 64%) and normal findings (55 of 85, 65%) had positive results, p=0·98. The study concluded that earlier maternal trimester of infection is associated with positive results but not clinical disease (p=0·04). ZIKV vertical transmission is frequent, but laboratory confirmed infection is not necessarily associated with infant abnormalities.
Research sample	The present study focuses on infants born to pregnant women enrolled in a cohort study at any week of gestation who presented to the acute febrile illness clinic at the Oswaldo Cruz Foundation with a rash that had developed within the previous 5 days and were found to be positive for ZIKV upon testing by RT-PCR at the time of rash. All live born infants of these women were eligible for enrollment. The longitudinal cohort was comprised of 244 pregnant women with confirmed ZIKV infection during pregnancy, of whom 223 (91·4%) had live births. Of these, 216 infants had clinical follow-up beyond birth. From this original cohort of 216 infants, 130 children (60%) had blood and or urine specimens obtained for ZIKV detection post-birth. The present report focuses on these 130 infants with clinical follow-up with laboratory diagnostic evaluations. The age at the time of performance of the first Zika diagnostic laboratory test ranged from birth to 148 days. Infants with repeat testing had an age ranging from 3 months to 397 days of age. The research sample of 130 infants was chosen because they were the children who had laboratory specimens available for testing. The population of 130 tested children was overall representative of the entire cohort of 216 children. There were no differences between frequency of abnormal findings overall, structural brain abnormalities, rates of abnormal neurosensory findings (eye and hearing exams) between both cohorts. All the children in the cohort with microcephaly (N=8) or who were small for gestational age (N=10) were in the group of 130 tested children. Below average neurodevelopment was more prevalent in the group of untested children.
Sampling strategy	This was a prospective cohort study of live infants born to mothers with a rash who had PCR-confirmed Zika virus infection during the Rio de Janeiro epidemic of 2015-2016. In that cohort 244 women were identified during the Rio epidemic; these were all pregnant women who came to the Fiocruz Febrile Illness Clinic with a rash that had developed in the last 5 days; all had a positive Zika virus PCR result in blood or urine the day they came to clinic. There were 10 fetal deaths and 11 women who were lost to follow-up before giving birth. As such there were 223 live births. Among those live births, 6 infants were lost to follow-up shortly after birth and 1 died in the second day of life. This was a sample of convenience as these were the patients identified prospectively during the ZIKV epidemic in Rio de Janeiro. the final sample presented in this article includes the 130 children (60%) had blood and or urine specimens obtained for ZIKV detection.
Data collection	The data was collected through standardized case report forms in real time by the study coordinator (Ms. Luana Damasceno and the clinical staff) and included patient interviews, medical history, physical examination findings and laboratory/ imaging/ and neurodevelopmental results. The data was collected by hand with pen and paper; our study coordinators collected the data during the time of each patient visit. As this was during and in the aftermath of the ZIKV epidemic, they were not blinded to the fact that infants were ZIKV exposed. Patients were first enrolled at the time of the ZIKV epidemic and followed subsequently over time. Infant serum and urine specimens were obtained following parental signed informed consent. Serum was collected by standard phlebotomy procedures and processed immediately for PCR while additional aliquots were stored at – 80C for subsequent testing for Zika IgM. Urine specimens collected by bagged urine collection, spun in a refrigerated centrifuge for 10 minutes, and the supernatant was aliquoted and processed immediately for PCR.
Timing	The start date for enrollment of infants was December 2015 to December 2016. There was no gap during the 13 month enrollment period.
Data exclusions	Participants without laboratory specimens available for testing were excluded. The rationale for exclusion is that 86 of 216 children did not have specimens available for diagnostic testing. As this was a novel disease at the time, testing for ZIKV was considered investigational and required IRB approval. Collection of specimens for ZIKV testing was initiated only after IRB approval was obtained and thus testing of children born earlier in the epidemic was less likely.
Non-participation	17 mothers enrolled with their infant and were lost to follow-up (11 before birth and 6 after birth). Because the mother was not able to be located for further follow-up, potential due to relocation, no reason was given for non-participation.
Randomization	Because there was no intervention, there was no need for randomization in this prospective cohort of infants. Covariates were measured and controlled for in analysis. Covariates were examined for colinearity and as potential confounders.

Reporting for specific materials, systems and methods

We require information from authors about some types of materials, experimental systems and methods used in many studies. Here, indicate whether each material, system or method listed is relevant to your study. If you are not sure if a list item applies to your research, read the appropriate section before selecting a response.

Materials & experimental systems

n/a	Involved in the study	
☐	☒	Antibodies
☒	☐	Eukaryotic cell lines
☒	☐	Palaeontology
☒	☐	Animals and other organisms
☐	☒	Human research participants
☒	☐	Clinical data

Methods

n/a	Involved in the study	
☒	☐	ChIP-seq
☒	☐	Flow cytometry
☒	☐	MRI-based neuroimaging

Antibodies

Antibodies used

ZIKV serologic testing of infants (IgM) was performed in duplicate serum aliquots using IgM antibody capture Zika MAC-ELISA from the Centers for Disease Control and Prevention (CDC, Fort Collins, CO, EUA).

Validation

CDC Zika MAC-ELISA as described in Granger, D. et al. Serologic Testing for Zika Virus: Comparison of Three Zika Virus IgM-Screening Enzyme-Linked Immunosorbent Assays and Initial Laboratory Experiences. J Clin Microbiol 2017; 55: 2127–36.
This assay received FDA EUA on 26 February 2016. Reagents for the CDC MAC-ELISA were provided to MDH, NYC DOHMH, and MML by the CDC, and select assay components required site-specific titrations. Prior to implementation of the CDC MAC-ELISA for clinical use, all centers passed a mandatory ZIKV serology panel (provided by the CDC) using the site-specific assay parameters. Briefly, 96-well high-affinity microtiter plates (Immulon 2 HB) were coated with goat anti-human IgM antibody (KPL, Gaithersburg, MD) at site-specific dilutions (1:2,000 at MDH and MML and 1:3,000 at NYC DOHMH) and incubated at 4°C overnight. Negative control (negative serum samples) and patient serum samples were used at a 1:400 dilution at all sites. A positive flavivirus IgM control was used at dilutions of 1:3,200 at MDH, 1:4,500 at NYC DOHMH, and 1:1,000 at MML. Controls and patient serum samples were added to a block of 6 wells and incubated for 1 h at 37°C. ZIKV antigen, either the Zika Vero E6 tissue culture antigen (MDH and NYC DOHMH) or Zika COS-1 recombinant antigen (MML), was added in triplicate to each patient and control block and incubated overnight at 4°C. Normal Vero E6 or COS-1 control antigens were used at the respective dilutions. Following washing, site-specific dilutions of the 6B6C-1 monoclonal antibody (1:2,000 at MDH, 1:1,000 at NYC DOHMH, 1:4,000 at MML), a chimeric monoclonal antibody specific for Flavivirus conjugated to horseradish peroxidase (MDH and NYC DOHMH purchased from Hennessey Research Associates, Shawnee, KS; MML purchased from InBios, Inc.), were added to the plates, which were further processed and analyzed per manufacturer recommendations. Results of the CDC MAC-ELISA were determined by calculating the ratio of the mean optical density (OD) of the patient sample (P) divided by the mean OD of the negative control (N), each reacted with the ZIKV antigen. P/N ratios of <2 were considered negative. Specimens with P/N ratios of ≥2 that showed background reactivity, evaluated by comparison of patient's OD value reacted with ZIKV antigen versus control antigen, were reported as inconclusive. Specimens with acceptable background reactivity levels and P/N ratios of ≥3 or between 2 and <3 were reported as presumptive positive or equivocal, respectively.

Human research participants

Policy information about studies involving human research participants

Population characteristics

see above

Recruitment

All children enrolled in this prospective observational cohort had in utero exposure to ZIKV. Their mothers were recruited for participation into a longitudinal ZIKV cohort during the Rio de Janeiro Zika virus epidemic of 2015-2016 (Brasil et al, NEJM Dec 2016). Pregnant women at any week of gestation who presented to the acute febrile illness clinic at the Oswaldo Cruz Foundation with a rash that had developed within the last 5 days were offered enrollment into the study. Those women with a positive PCR result for ZIKV in blood or urine were enrolled into the prospective Zika cohort. Live born infants to mothers enrolled in the prospective ZIKV cohort comprise the infants followed in the present study. They all had PCR-confirmed ZIKV in utero exposure. Recruitment site was the Fiocruz Foundation medical facilities in Rio de Janeiro for mothers and their infants. Because of the nature of maternal inclusion criteria we only enrolled infants who were born to mothers with symptomatic ZIKV infection during pregnancy (all women had a rash, and this is why they came to medical attention). Infants were not recruited because mothers had abnormal ultrasounds or there were abnormalities at birth. All children included in the present study had to be enrolled antenatally to a mother who had a rash during pregnancy in the last 5 days and was found to have a positive PCR for ZIKV in blood or urine. Researchers in the study were not blinded to the apparent clinical abnormalities of children who were born with microcephaly and congenital ZIKV syndrome or who were small for gestational age. Children who had visible abnormal clinical features at the time of birth could have been more likely to have been tested early in life, which could lead to a selection bias in testing. Overall however, the frequency of any abnormal clinical finding in both tested and untested children turned out to be the same 35% in tested children versus 38% in untested children, p = 0.70.

Ethics oversight

The study protocol was approved by the institutional review boards at Fundação Oswaldo Cruz (Fiocruz) and the University of California, Los Angeles. Participants provided written informed consent. The authors vouch for the accuracy and completeness of the data and the analyses and for the fidelity of the study to the protocol.

Note that full information on the approval of the study protocol must also be provided in the manuscript.
